# Supplementary material for: RNA elements required for the high efficiency of West Nile Virus-induced ribosomal frameshifting
Source: bioRxiv. 2024 Oct 16:2024.10.16.618579. Preprint. [Version 1] doi: 10.1101/2024.10.16.618579 (PMC11507841; doi:10.1101/2024.10.16.618579)
Supplement: Supplement 1 [file media-1.pdf]

Supplementary Information

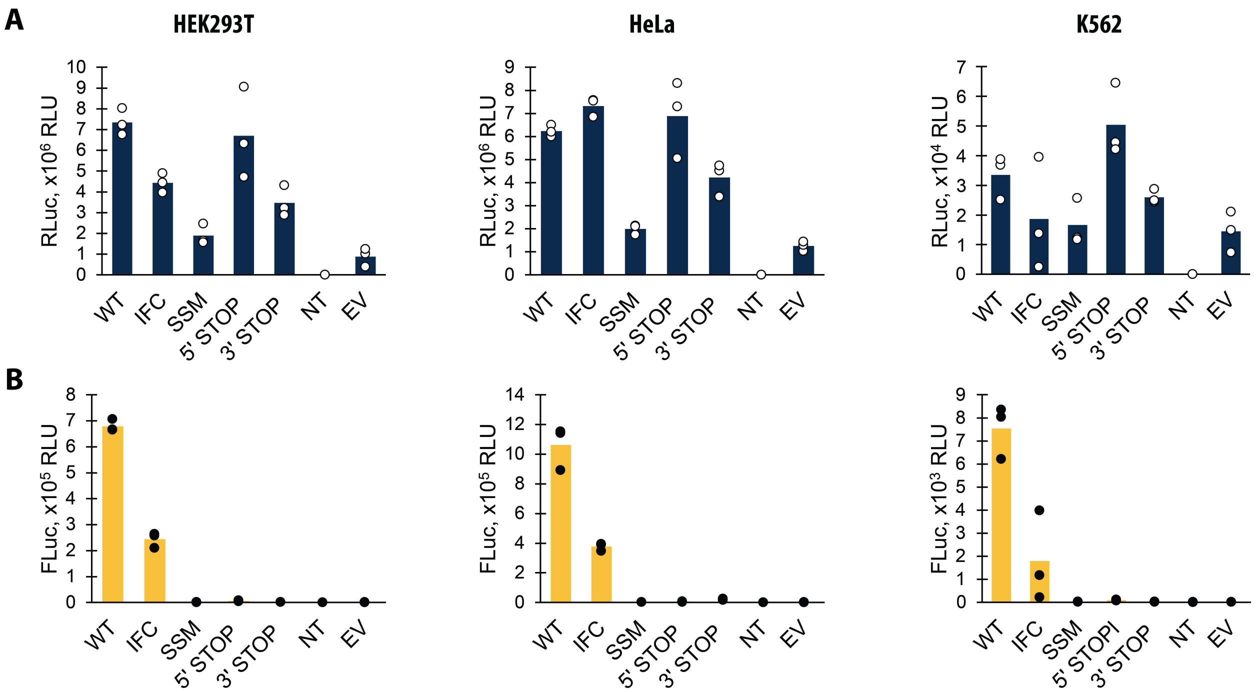

Figure S1. Measurement of Renilla and Firefly luciferase activities in frameshifting reporter assays across different cell lines.

(**A**) Renilla luciferase (RLuc) activities of the wild-type (WT) and mutant reporter constructs measured in HEK293T, HeLa, and K562 cell lines.

(**B**) Firefly luciferase (FLuc) activities measured from the same reporter constructs across the three cell lines. The WT and IFC constructs show high FLuc activity, with the IFC demonstrating efficient translation in the 0 frame. In contrast, the SSM, 5′ STOP, 3′ STOP, and EV constructs show minimal FLuc activity, consistent with impaired translation due to mutations or stop codon introduction at the frameshifting site.

Bars represent the means of three independent experiments, with each dot corresponding to the individual experimental data points.

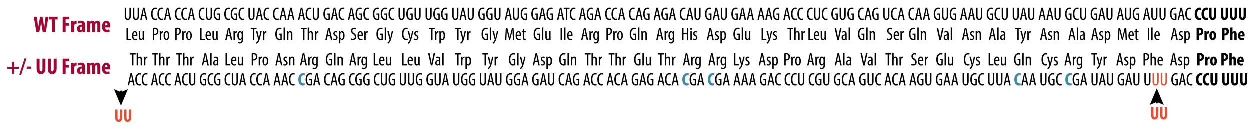

Figure S2. Detailed representation of the nucleotide and amino acid sequences of the wild-type (WT) and mutant UU frameshifting constructs.

This figure shows the nucleotide and corresponding amino acid sequences of the wild-type (WT) and mutant frameshifting constructs. In the mutant construct, two nucleotides (UU) were deleted near the 5′ end of the sequence and reinserted closer to the slippery site (marked with arrows). The slippery site is shown in bold. Five nucleotide changes made to remove stop codons that appeared after the UU deletion are shown in blue and bolded.

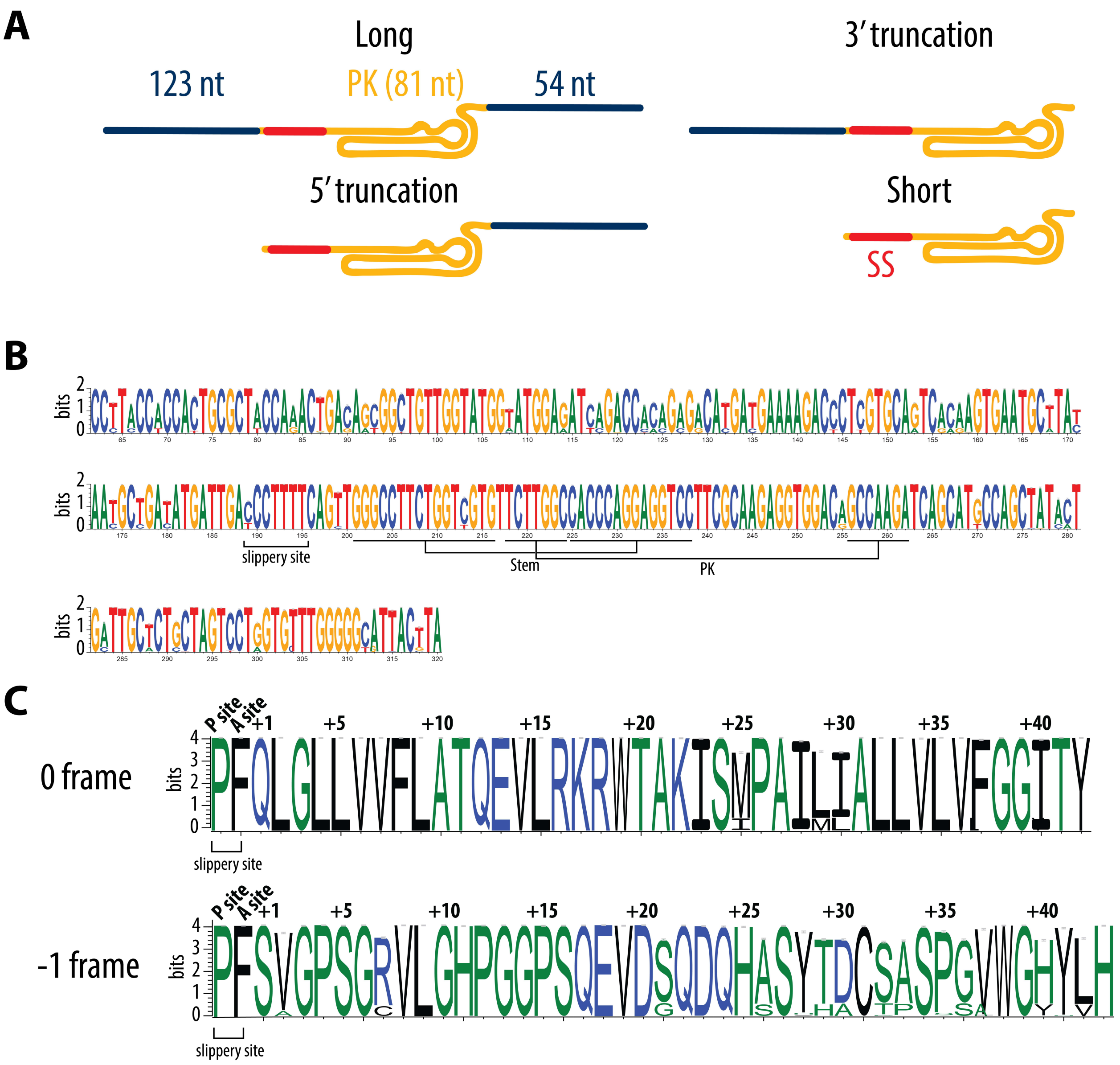

Figure S3. Detailed schematic of truncation constructs and sequence conservation at the WNV PRF site.

(**A**) Schematic representation of the truncation constructs related to **Figure 2D**. The blue regions indicate the flanking sequences with numbers of nucleotides, the golden regions represent the pseudoknot (PK) structure, and the red marks show the slippery site (SS). The "Long" construct includes the full-length flanking sequences and pseudoknot. The "5′ truncation" and "3′ truncation" constructs progressively reduce the flanking sequences, while the "Short" construct contains minimal flanking sequences with the pseudoknot and slippery site intact.

(**B**) Information content in bits of nucleotides upstream and downstream from the WNV programmed -1 ribosomal frameshifting (PRF) site were calculated using WebLogo3 for all complete WNV genomes deposited in the NCBI database. The height of each position represents

the relative information content of each position in bits and the x-axis displays the relative position of the nucleotide in the multiple sequence alignment. The slippery site and nucleotides involved in pseudoknot formation are indicated. The sequence logo illustrates the degree of conservation for each nucleotide position, highlighting key elements necessary for frameshifting and predicted pseudoknot stability.

(**C**) Information content in bits of the translated polypeptides downstream from the frameshifting site in both the 0 frame and -1 frame calculated using WebLogo3 for all complete WNV genomes deposited on NCBI. The height of each position represents the relative information content of each position in bits and the x-axis displays the relative position of the amino acid in the multiple sequence alignment. The slippery site is indicated, and the sequence logo shows the conservation level of each amino acid in the two reading frames, providing insights into the potential variability of translation products depending on frameshifting events. Codon 44 is a stop codon.

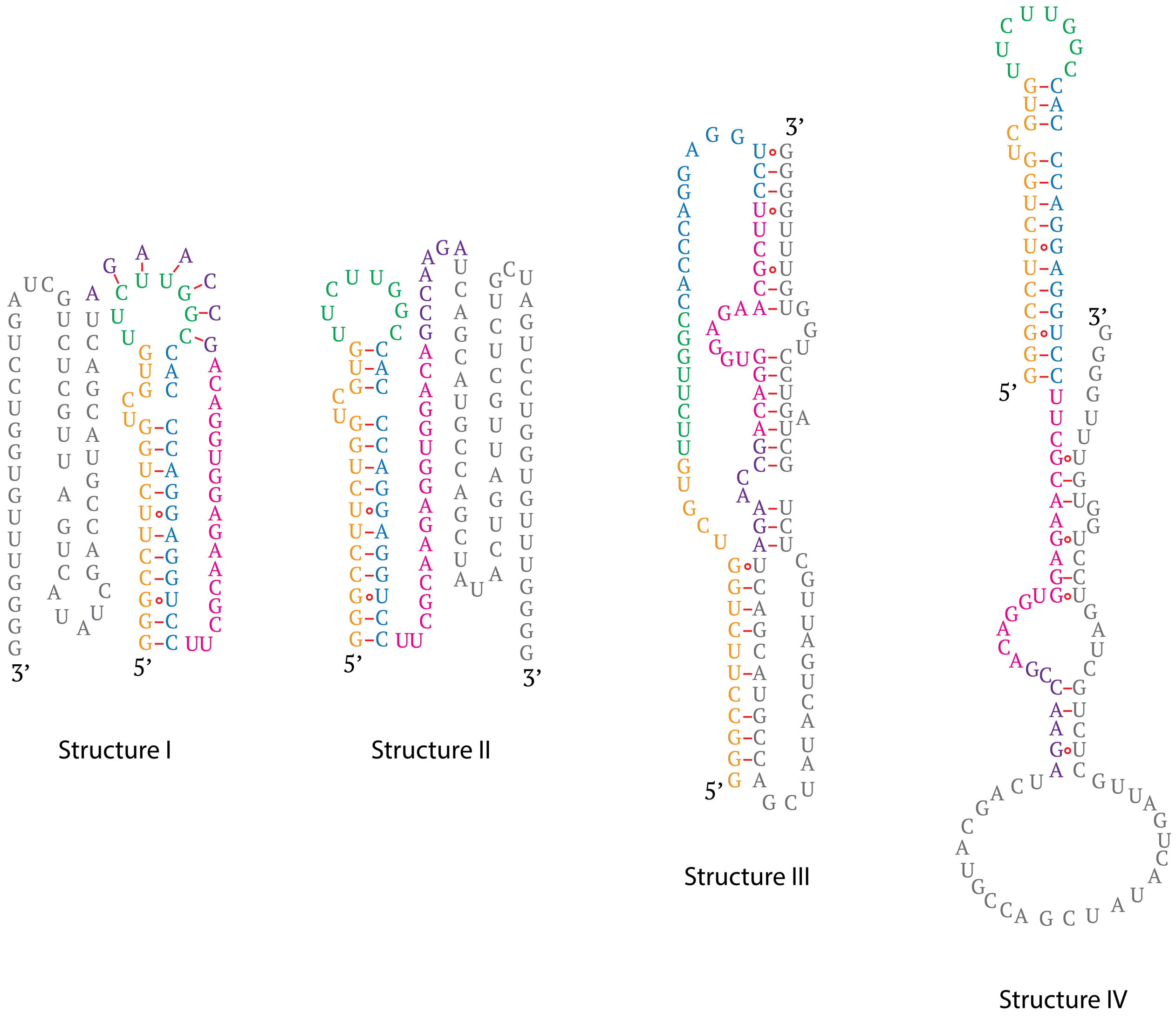

Figure S4. Predicted secondary structures of the West Nile Virus (WNV) programmed -1 ribosomal frameshifting (PRF) site. Color coding is used for the tracking of how different sequences are involved in the formation of the alternative secondary structures. Secondary structures were generated based on the data in (27).

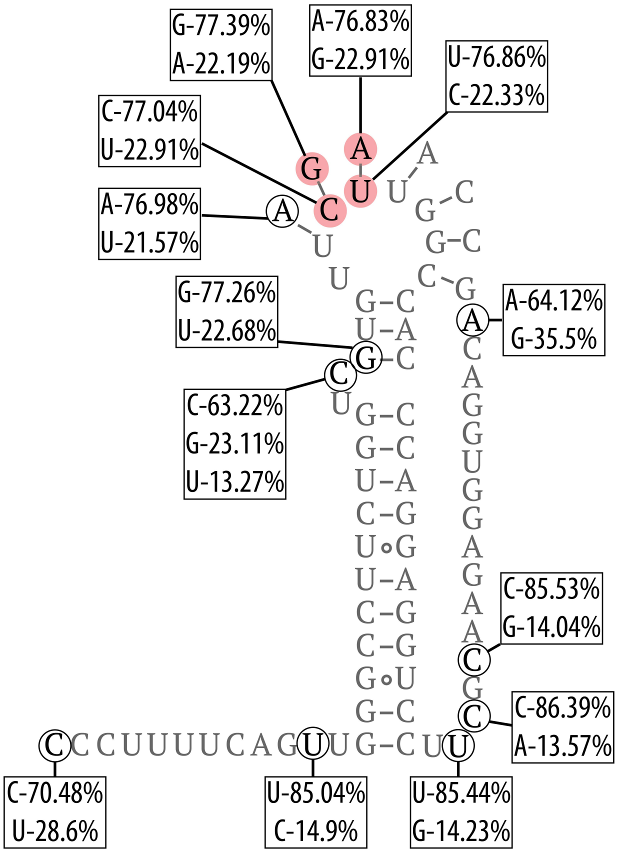

Figure S5. Detailed analysis of nucleotide variation mapped onto the predicted secondary structure of the WNV pseudoknot. The red-highlighted nucleotide pairs show concerted compensatory changes in the sequence consistent with the proposed secondary structure, though the statistical significance of these changes is low. These co-occurring changes support the WNV pseudoknot structure and are consistent with a conserved fold.

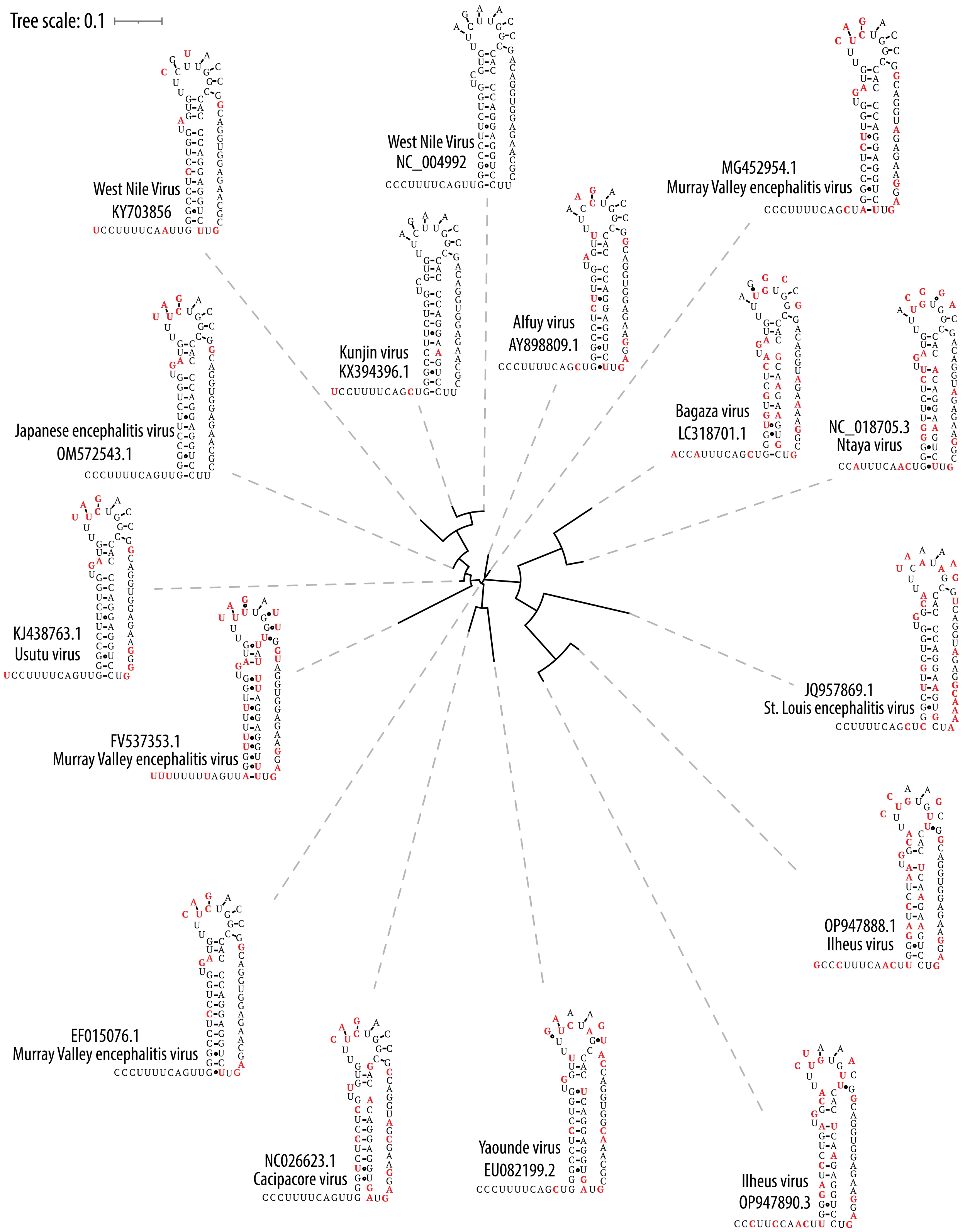

Figure S6. Maximum-likelihood tree of *Flavivirus* frameshifting elements. Maximum-likelihood tree of *Flavivirus* frameshifting elements with representative secondary structures. Sequence variations from the strain NY99 (RefSeq NC_009942) are colored in red.

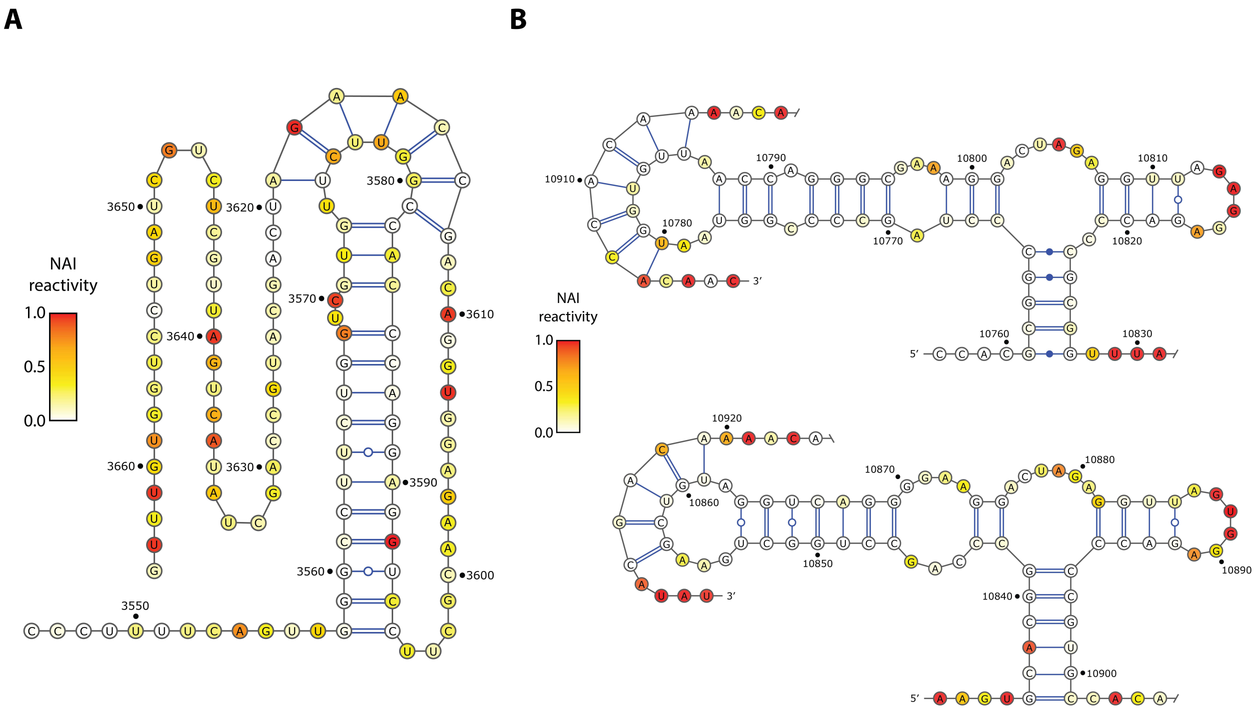

Figure S7. Analysis of the predicted secondary structure and nucleotide reactivity of the WNV PRF element.

(**A**) NAI reactivity profiling of the WNV PRF element from WNV-infected Vero cells mapped onto the proposed secondary structure. The downstream nucleotides exhibit elevated levels of NAI reactivity, indicating the absence of a stable secondary structure. The pseudoknot nucleotides show somewhat elevated reactivity levels, suggesting that the pseudoknot might possess conformational flexibility rather than being fully rigid.

(**B**) NAI reactivity profiling of two well-characterized dumbbell pseudoknot structures in the 3′ untranslated region (UTR) of WNV from WNV-infected Vero cells shown for comparison. These structures display much lower reactivity levels in known base-paired regions, reflecting the stability of their secondary structures in the same experiment. This comparison highlights how reactivity patterns likely correlate with structural stability in pseudoknots.

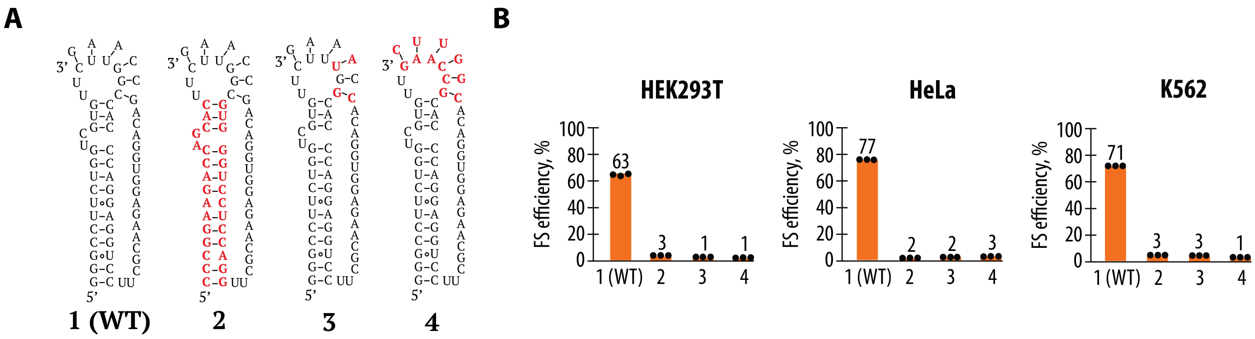

Figure S8. Compensatory mutations aimed at restoring the predicted pseudoknot structure. (**A**) Schematic representation of the compensatory mutations. (**B**) Effects of the compensatory mutations on frameshifting in HEK293T, HeLa, and K562 cells, compared to the wild-type (WT) construct. Bars represent the means of three independent experiments, with each dot corresponding to the individual experimental data points.

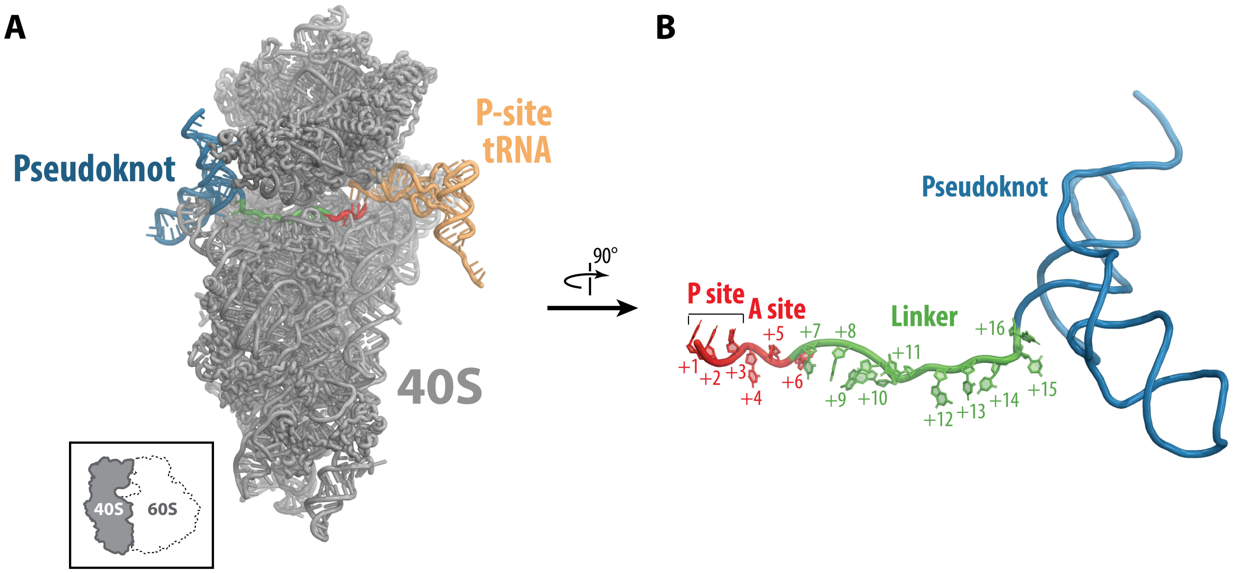

Figure S9. Cryo-EM structure of the SARS-CoV-2 frameshifting site. (**A**) Overview of the rabbit ribosome structure bound to mRNA containing the pseudoknot from SARS-CoV-2 (PDB ID: 7o7z). The 40S subunit is shown in gray, the P-site tRNA in orange, the pseudoknot in blue, the codons of the A- and P-sites in red, and the linker between the pseudoknot and the A- and P-site codons in green. The inset shows the 60S subunit removed for clarity, leaving only the 40S subunit shown in a side view. (**B**) Close-up view of the mRNA structure. The same color coding is used, with the 40S subunit not shown for clarity. The nucleotide numbering starts from the first nucleotide of the P-site codon (+1) and continues to the last unpaired nucleotide (+16). The view is from the solvent side of the 40S subunit in panel (**A**).

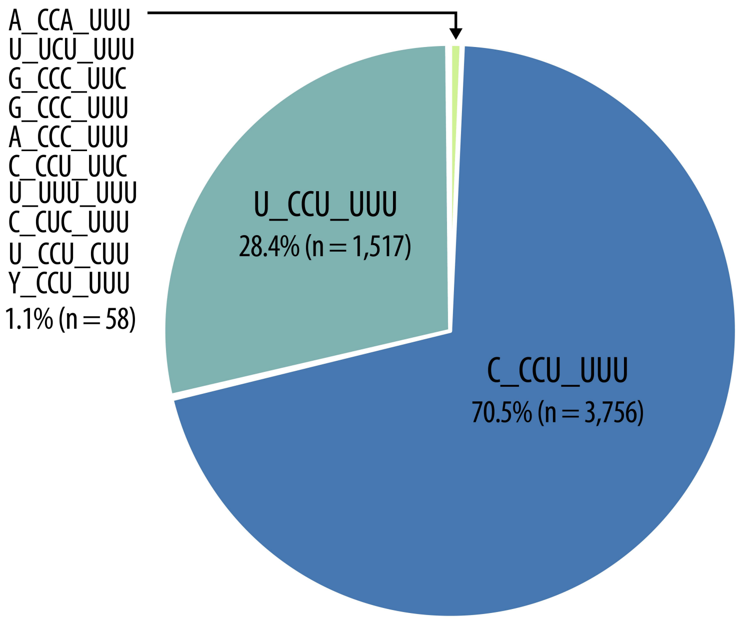

Figure S10. Distribution of slippery sequences among flaviviruses. The chart displays the proportion of different slippery sequences. The number of genomes and the corresponding percentages of the total are indicated for each sequence variant.

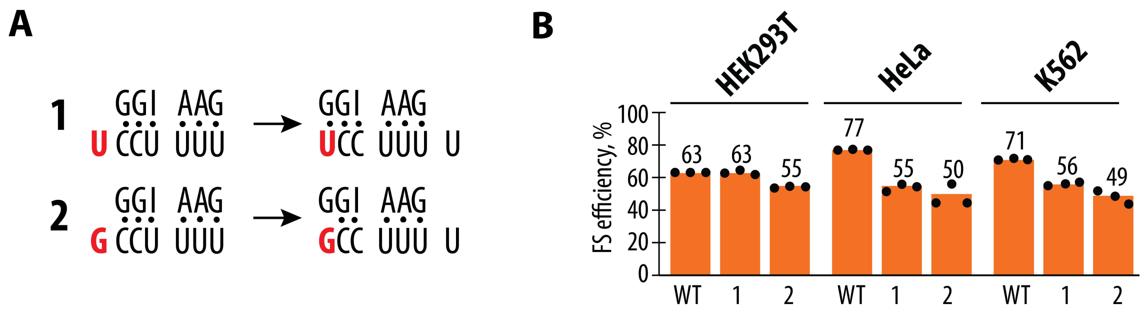

Figure S11. Effect of the first nucleotide in the slippery site on frameshifting efficiency. (**A**) Mutational analysis of the first nucleotide in the slippery site, with sequence register before (frame 0, left) and after (frame -1, right) frameshifting. (**B**) Frameshifting efficiency of constructs with mutations of the first nucleotide of slippery site, in HEK293T, HeLa, and K562 cells. Frameshifting efficiency is shown as a percentage of the ratio between normalized FLuc/RLuc luminescence in the -1 frame to the sum of normalized Fluc/Rluc luminescence in the -1 and 0 frames. Bars represent the means of three independent experiments, with each dot corresponding to the individual experimental data points.

Supplementary Table S1. Synthetic DNA blocks used in this study

Figure	DNA name	Sequence
1B	WT	TCGAGTCCAACCCCGGGCCCTACTCGAGCTTACCACCACTGCGCT<br>ACCAAACTGACAGCGGCTGTTGGTATGGTATGGAGATCAGACCAC<br>AGAGACATGATGAAAAGACCCTCGTGCAGTCACAAGTGAATGCT<br>TATAATGCTGATATGATTGACCCTTTTCAGTTGGGCCTTCTGGTCG<br>TGTTCTTGGCCACCCAGGAGGTCCTTCGCAAGAGGTGGACAGCCA<br>AGATCAGCATGCCAGCTATACTGATTGCTCTGCTAGTCCTGGTGTT<br>TGGGGGCATTACTTACGATCTGAGGCACGGCATAAGCAAAAGAT<br>CGTAG
	IFC	TCGAGTCCAACCCCGGGCCCTACTCGAGCTTACCACCACTGCGCT<br>ACCAAACTGACAGCGGCTGTTGGTATGGTATGGAGATCAGACCAC<br>AGAGACATGATGAAAAGACCCTCGTGCAGTCACAAGTGAATGCT<br>TATAATGCTGATATGATTGACCCTTTTCAGTTGGGCCTTCTGGTCG<br>TGTTCTTGGCCACCCAGGAGGTCCTTCGCAAGAGGTGGACAGCCA<br>AGATCAGCATGCCAGCTATACTGATTGCTCTGCTAGTCCTGGTGTT<br>TGGGGGCATTACTTACAGATCTGAGGCACGGCATAAGCAAAAGA<br>TCGTA
	SSM	TCGAGTCCAACCCCGGGCCCTACTCGAGCTTACCACCACTGCGCT<br>ACCAAACTGACAGCGGCTGTTGGTATGGTATGGAGATCAGACCAC<br>AGAGACATGATGAAAAGACCCTCGTGCAGTCACAAGTGAATGCT<br>TATAATGCTGATATGATTGATCGTACTCAGTTGGGCCTTCTGGTCG<br>TGTTCTTGGCCACCCAGGAGGTCCTTCGCAAGAGGTGGACAGCCA<br>AGATCAGCATGCCAGCTATACTGATTGCTCTGCTAGTCCTGGTGTT<br>TGGGGGCATTACTTACGATCTGAGGCACGGCATAAGCAAAAGAT<br>CGTAG
	5' STOP	TCGAGTCCAACCCCGGGCCCTACTCGAGCTAATTACCACCACTGC<br>GCTACCAAACTGACAGCGGCTGTTGGTATGGTATGGAGATCAGAC<br>CACAGAGACATGATGAAAAGACCCTCGTGCAGTCACAAGTGAAT<br>GCTTATAATGCTGATATGATTGACCCTTTTCAGTTGGGCCTTCTGG<br>TCGTGTTCTTGGCCACCCAGGAGGTCCTTCGCAAGAGGTGGACAG<br>CCAAGATCAGCATGCCAGCTATACTGATTGCTCTGCTAGTCCTGG<br>TGTTTGGGGGCATTACTTACGATCTGAGGCACGGCATAAGCAAAA<br>GATCG
2A	Mutant NC<br>WT	GGCGGGAGACGTCGAGTCCAACCCCGGGCCCTACTCGAGCACCA<br>CCACTGCGCTACCAAACCGACAGCGGCTGTTGGTATGGTATGGAG<br>ATCAGACCACAGAGACACGACGAAAAGACCCTCGTGCAGTCACA<br>AGTGAATGCTTACAATGCCGATATGATTTTGACCCTTTTCAGTTGG<br>GCCTTCTGGTCGTGTTCTTGGCCACCCAGGAGGTCCTTCGCAAGA<br>GGTGGACAGCCAAGATCAGCATGCCAGCTATACTGATTGCTCTGC

		TAGTCCTGGTGTTTGGGGGCATTACTTACGATCTGAGGCACGGCATAAGCAAAAGATCGTAGCCCCAGTAAAGCAAACACTCAACTTCGATCTACTCAAACTCGCAGGTGATGTGGAATCTAATCCAGGACCTTTCGGATCTGCCACCATTGAAGATGCCAAAAACATTAAGAAGGGCCCAGCGCCATTCTACCCACTCGAAGACGG
	Mutant NC IFC	GGCGGGAGACGTCGAGTCCAACCCCGGGCCCTACTCGAGCACCACCACTGCGCTACCAAACCGACAGCGGCTGTTGGTATGGTATGGAGATCAGACCACAGAGACACGACGAAAAGACCCTCGTGCAGTCACAAGTGAATGCTTACAATGCCGATATGATTTTGACCCTTTTCAGTTGGGCCTTCTGGTCGTGTTCTTGGCCACCCAGGAGGTCCTTCGCAAGAGGTGGACAGCCAAGATCAGCATGCCAGCTATACTGATTGCTCTGCTAGTCCTGGTGTTTGGGGGCATTACTTACAGATCTGAGGCACGGCATAAGCAAAAGATCGTAGCCCCAGTAAAGCAAACACTCAACTTCGATCTACTCAAACTCGCAGGTGATGTGGAATCTAATCCAGGACCTTTCGGATCTGCCACCATTGAAGATGCCAAAAACATTAAGAAGGGCCCAGCGCCATTCTACCCACTCGAAGACGG
5B	-2 reporter WT	GGCGGGAGACGTCGAGTCCAACCCCGGGCCCTACTCGAGCTTACCACCACTGCGCTACCAAACTGACAGCGGCTGTTGGTATGGTATGGAGATCAGACCACAGAGACATGATGAAAAGACCCTCGTGCAGTCACAAGTGAATGCTTATAATGCTGATATGATTGACCCTTTTCAGTTGGGCCTTCTGGTCGTGTTCTTGGCCACCCAGGAGGTCCTTCGCAAGAGGTGGACAGCCAAGATCAGCATGCCAGCTATACGCACGGCATAAGGTAAAGCAAACACTCAACTTCGATCTACTCAAACTCGCAGGTGATGTGGAATCTAATCCAGGACCTTTCGGATCTGCCACCATTGAAGATGCCAAAAACATTAAGAAGGGCCCAGCGCCATTCTACCCACTCGAAGACGG
	-2 reporter IFC	GGCGGGAGACGTCGAGTCCAACCCCGGGCCCTACTCGAGCTTACCACCACTGCGCTACCAAACTGACAGCGGCTGTTGGTATGGTATGGAGATCAGACCACAGAGACATGATGAAAAGACCCTCGTGCAGTCACAAGTGAATGCTTATAATGCTGATATGATTGACCCTTTTCAGTTGGGCCTTCTGGTCGTGTTCTTGGCCACCCAGGAGGTCCTTCGCAAGAGGTGGACAGCCAAGATCAGCATGCCAGCTATACGCACGGCAGTAAAGCAAACACTCAACTTCGATCTACTCAAACTCGCAGGTGATGTGGAATCTAATCCAGGACCTTTCGGATCTGCCACCATTGAAGATGCCAAAAACATTAAGAAGGGCCCAGCGCCATTCTACCCACTCGAAGACGG

Supplementary Table S2. Primers used in this study

Figure	Primer name	Sequence
1B	3' control PCR Fwd	TACGATCTTTTGCTTATGCCGTGCC
	3' control PCR Rev	AAGCAAACACTCAACTTCGATCTAC
	3' control Gibson insert	GAGGCACGGCATAAGCAAAAGATCGTAGCC<br>TAAGTAAAGCAAACACTCAACTTCGATCTA
1D	Vector PCR Fwd	ACCATACCCATACGACGTACCAGATTACGCTTAGCGT<br>CTTCACACTCGAAGATTTCGTTG
	Vector PCR Rev	CAGTTTGGTAGCGCAGCAGTGGTAAGCCACCAGAGCCC<br>ATATGCCCTCCA
	Insert PCR Fwd	TGGAGGGCATATGGGCTCTGGTGGCTTACCACCACTGCGC<br>ATCCAACTGCGCTACCAAACTG
	Insert PCR Rev	GCTAAGCGTAATCTGGTACGTCGTATGGGTATGGTAAGTAA<br>TGCCCCCAAACACCAGG
2D	5' truncation PCR FWD	GCTCGAGTAGGGCCC
	5' truncation PCR Rev	GACCCTTTTCAGTTGGGC
	3' truncation WT PCR FWD	GTATAGCTGGCATGCTG
	3' truncation WT PCR Rev	GATCTGAGGCACGGC
	3' truncation IFC PCR FWD	GTATAGCTGGCATGCTG
	3' truncation IFC PCR Rev	AGATCTGAGGCACGGC

	Short insert PCR WT1	GAGTCCAACCCCGGGCCCTACTCGAGCGACCCTTTTCAGTTGGGC CTTCTGGTCGTGTTC
	Short insert PCR WT2	CAGTTGGGCCTTCTGGTCGTGTTCTTGGCCACCCAGGAGGTC CTTCGCAAGAGGTGGAC
	Short insert PCR WT3	TGCTTATGCCGTGCCTCAGATCGCTGATCTTGGCTGTCCACC TCTTGCGAAGGACCTCCT
	Short insert PCR IFC1	GAGTCCAACCCCGGGCCCTACTCGAGCGACCCTTTTCAGT TGGGCCTTCTGGTCGTGTTC
	Short insert PCR IFC2	CAGTTGGGCCTTCTGGTCGTGTTCTTGGCCACCCAGGAGGTC CTTCGCAAGAGGTGGAC
	Short insert PCR IFC3	TGCTTATGCCGTGCCTCAGATCTGCTGATCTTGGCTGTCCACC TCTTGCGAAGGACCTCC
3A	Construct 2 Fwd	ACCAGCACTTCTTGGCCACCCAG
	Construct 2 Rev	CTTCCGGGAACTGAAAAGGGTCAATCATATC
	Construct 3 Fwd	CTCCAGGTTCGCAAGAGGTGGAC
	Construct 3 Rev	GACCCACGCCAAGAACACGACC
	Construct 4 Fwd	TCCACCTGTGCCAAGATCAGCATGC
	Construct 4 Rev	GAACGCTTGGACCTCCTGGGTGGG
	Construct 5 Fwd	GTGTTCTTTGGCACCCAGGAGGTCCTTC
	Construct 5 Rev	GACCAGAAGGCCCAACTGAAAAGGGTC
	Construct 6 Fwd	TGGTCGTGTTGAACCHCACCCAGGAG

	Construct 6 Rev	GAAGGCCCAACTGAAAAG
4A	2 nt linker PCR Fwd	GGGCCTTCTGGTCGTG
	2 nt linker PCR Rev	TGAAAAGGGTCAATCATATCAGC
	8 nt linker PCR Fwd	TTTGGGCCTTCTGGTCGTG
	8 nt linker PCR Rev	AACTGAAAAGGGTCAATCATATCAG
	11 nt linker PCR Fwd	TTTAAAGGGCCTTCTGGTCGTG
	11 nt linker PCR Rev	AACTGAAAAGGGTCAATCATATCAG
4C	Construct 2 PCR Fwd	GAAGGCGGAACTGAAAAGGGTCAATCATATC
	Construct 2 PCR Rev	TGGTCGTGTTCTTGGCCACCCAG
	Construct 3 PCR Fwd	GGTCGTGTTCTTGGCCACCCAGGAG
	Construct 3 PCR Rev	AGAAGGGGGAACYGAAAAGGGTCAATCATATC
	Construct 4 PCR Fwd	GGTCGTGTTCTTGGCCACCCAGGAG
	Construct 4 PCR Rev	AGAAGCGGGAACTGAAAAGGGTCAATCATATC
	Construct 5 PCR Fwd	TGGTCGTGTTCTTGGCCACCCAG
	Construct 5 PCR Rev	GAACCGGGAACTGAAAAGGGTCAATCATATC
	Construct 6 PCR Fwd	TGGTCGTGTTCTTGGCCACCCAG

	Construct 6 PCR Rev	CTTCCGGGAACTGAAAAGGGTCAATCATATC
5B	U_UUU_UUU slippery site PCR Fwd	TTTTCAGTTGGGCCTTCTG
	U_UUU_UUU slippery site PCR Rev	AAATCAATCATATCAGCATTATAAGC
6B	P1 Fwd	TTTTCAGTTGGGCCTTCTG
	P1 Rev	GCGTCAATCATATCAGCATTATAAGC
	P2 Fwd	TTTTCAGTTGGGCCTTCTG
	P2 Rev	CGGTCAATCATATCAGCATTATAAGC
	P3 Fwd	GGGTCAATCATATCAGCATTATAAGC
	P3 Rev	GTTTCAGTTGGGCCTTCTG
	P4 Fwd	ATTTCAGTTGGGCCTTCTG
	P4 Rev	GGGTCAATCATATCAGCATTATAAGC
	P5 Fwd	CTTTCAGTTGGGCCTTCTG
	P5 Rev	GGGTCAATCATATCAGCATTATTAGC
6D	A1 Fwd	AAAACAGTTGGGCCTTCTG
	A1 Rev	GGGTCAATCATATCAGCATTATAAGC
	A2 Fwd	TTTCCAGTTGGGCCTTCTG
	A2 Rev	GGGTCAATCATATCAGCATTATAAGC
	A3 Fwd	TGTTCAGTTGGGCCTTCTG
	A3 Rev	GGGTCAATCATATCAGCATTATAAGC
	A4 Fwd	TTGTCAGTTGGGCCTTCTG
	A4 Rev	GGGTCAATCATATCAGCATTATAAGC
	A5 Fwd	TTTGCAGTTGGGCCTTCTG
	A5 Rev	GGGTCAATCATATCAGCATTATAAGC

S8	Construct 2 PCR FWD	TTGGCGTGGGTCCTCCAGGTTCGCAAGAGGTGGAC
	Construct 2 PCR Rev	GAAGTGCTGGTCTTCCGGGAACTGAAAAGGGTCAATCATATC
	Construct 3 PCR FWD	GTGTTCTTTGGCACCCAGGAGGTCCTTC
	Construct 3 PCR Rev	GACCAGAAGGCCCAACTGAAAAGGGTC
	Construct 4 PCR FWD	CGGTTCATCAGCATGCCAGCTATAC
	Construct 4 PCR Rev	TGTCCACCTCTTGCGAAGGAC
S10	Construct 1 PCR FWD	TTTTCAGTTGGGCCTTCTG
	Construct 1 PCR Rev	GGATCAATCATATCAGCATTATAAGC
	Construct 2 PCR FWD	TTTTCAGTTGGGCCTTCTG
	Construct 2 PCR Rev	GGCTCAATCATATCAGCATTATAAGC
